# How do mature non-graduate students compare to the rest of the cohort in medical training? A UKMED study

**DOI:** 10.1186/s12909-026-09776-6

**Published:** 2026-06-26

**Authors:** Nana Sartania, Adam Troughton, Philip Chan

**Affiliations:** 1 School of Medicine Dentistry and Nursing, College of Medical, Veterinary and Life Sciences, University of Glasgow, Glasgow, G12 8QQ United Kingdom; 2 General Medical Council, 3 Hardman Street, Manchester, M3 3AW United Kingdom; 3 Kent and Medway Medical School, Canterbury Christ Church University and University of Kent, Canterbury, CT2 7FS United Kingdom

**Keywords:** Widening Participation, Differential attainment, Specialty choice, Undergraduate and Postgraduate Outcome Measures, Socio-economic disadvantage

## Abstract

**Background:**

Widening participation in medicine is a key societal priority. To improve representation of non-traditional applicants, UK medical schools use contextual admissions, although definitions of under-represented groups vary across institutions. This study examined the educational and training trajectories of one such group—*mature non-graduates*. We aimed to determine whether their progression was comparable to that of school-leavers and graduate entrants, whether they progressed through medical school non-inferiorly, and whether they were equally likely to secure postgraduate training posts.

**Methods:**

Data on UK medical students and resident doctors (2007–2015) were extracted from the UK Medical Education Database (UKMED). Undergraduate performance (Educational Performance Measure (EPM), Prescribing Safety Assessment (PSA), Foundation Programme Situational Judgement Test (FP-SJT) and postgraduate performance (Membership of the Royal College of Physicians, MRCP and MRCGP Applied Knowledge Test, MRCGP AKT), Annual Review of Competency Progression (ARCP), and obtaining an offer for a Level 1 training post) were compared across the three groups using multivariate statistical analyses, including Kruskal–Wallis tests where appropriate.

**Results:**

Across all measures of undergraduate academic attainment (EPM, PSA and FP-SJT) and postgraduate examination performance (MRCP Part 1, Part 2 and PACES), mature non-graduates performed less well than school-leavers or graduates. They were more frequently released from training (ARCP Outcome 4) and received higher numbers of Developmental ARCP outcomes. Mature non-graduates were also less likely to receive an offer for competitive specialty training on their first application; however, the likelihood of them applying exclusively to General Practice did not differ from that of school-leavers or graduates.

**Conclusions:**

Although performance differences persist for a subset of widening-participation candidates named here as mature non-graduates, they still progress through training, obtain Certificates of Completion of Training, and contribute to the medical workforce. Further research is needed to examine how their life experience and non-traditional educational trajectories influence their long-term practice.

**Supplementary Information:**

The online version contains supplementary material available at https://doi.org/10.1186/s12909-026-09776-6.

## Background

It has been stated that equity is a priority theme in the higher education agenda of governments – of the UK [1, 2] and of many other countries. [3] The paucity of social mobility is widely discussed in recent academic publications. [4, 5] A review of the HE equity policies globally has found that only a tiny minority (1%) of the poorest young adults complete at least four years of higher education, compared to 20% of the richest. [6] The picture is even grimmer for highly selective courses such as medicine. According to the Universities and Colleges Admissions Service in the UK (UCAS), over a third of the 18-year-olds (36%) in the country pursue higher education, [7] but of those who enter higher education from the most deprived areas, few have historically joined medical schools, e.g. only 6% in 2014. A decade later, the number of entrants from Index of Multiple Deprivation Quintile 1 (IMD Q1, the most deprived areas), has more than doubled to 14% as a result of a concerted effort from the UK Government and the Higher Education sector, in a wave of sustained initiatives to expand access to medical education to historically underrepresented groups, via contextual admissions and outreach. [8, 9] Currently, as part of efforts to widen participation, most UK medical schools employ contextual admissions to lower entry requirement for students from socio-economically disadvantaged backgrounds. [10] These students may be admitted directly into year 1 of the Medicine course or, if their prior attainment is insufficient for direct entry, enrol in a Gateway programme—a one-year pre-medicine course that provides additional academic and pastoral support. [11, 12] This approach has been complemented by a range of outreach initiatives led by universities, Royal Colleges and charitable organizations such as *The Sutton Trust*, *In2Science*, *So You Want to Be a Doctor*, and *The Social Mobility Foundation*.

Efforts to address the gender and ethnicity gap in medical schools began gaining a renewed momentum in the mid-20th century and have intensified globally in recent years. Although gender parity among medical students has largely been achieved, significant inequalities persist in senior leadership, pay, and clinical research. [13, 14] In contrast, substantial socio-economic disparity in access to medical education continue to endure and research examining the achievements, progression and professional outcomes of students from disadvantaged backgrounds remains sorely lacking.

In this paper, we examine the progression of individuals from lower socio-economic backgrounds throughout their medical training and explore their subsequent specialty destinations, with the aim of identifying pertinent issues relevant to widening participation (WP) beyond the initial admission to medical schools.

It has been suggested that doctors from WP backgrounds are more likely to apply for general practice training than their non-WP peers. [2, 15] When looking at factors associated with resident doctors’ decision to apply exclusively to GP training programmes, Gale et al., [15] identified the Index of Multiple Deprivation (IMD), the National Statistics Socio-economic classification (NS-SEC), [16] and ethnicity as significant predictors. However, it remained unclear whether this was generally a positive choice for General Practice or a default position due to a lack of access to the more competitive specialties, so more analysis was needed on not just the specialty destinations of WP students but also their progress and achievements in the undergraduate years. GMC data on uptake for specialty training posts in the last decade indicate marked differences in demand between general practice and surgical training programmes. Surgical training was particularly competitive in early 2000, with application rates fluctuating around 2.1-3, [17] whereas the competition ratio for GP training programmes at the time remained relatively stable at approximately 1.2–1.4 [18]. This high level of competition for surgical training, together with evidence suggesting that students from widening participation backgrounds are less likely to pursue surgery as a career destination, [19, 20] we considered it important to interrogate our dataset by focusing specifically on GP and surgery as contrasting specialty destinations.

A major impediment to research into this area is the lack of a standardised definition of widening participation. Even within the UK, where the WP discourse is largely focused on social class, there is no agreed definition, and the individual medical schools use very different definitions from each other and from government. This creates confusion in the numbers or WP students admitted, and in turn some uncertainty about the claims of progress and the effectiveness of various initiatives. Indeed, it is genuinely difficult to define any single criterion as encompassing the whole complexity of widening participation as many different factors may contribute to disadvantage. Therefore, as recommended by the Medical Schools Council, many schools apply multiple criteria to define several WP categories, each arguably underrepresented and targeted for greater inclusion. Criteria thus vary but may include factors such as residential postcode to identify applicants from the most deprived areas; receipt of free school meals (FSM) or eligibility for means-tested bursaries (e.g., the UCAT bursary); care experience (e.g. ‘young carer’ status); refugee/asylum seeker status; armed services families and many more. This approach results in much variation in WP definitions between schools, research academics, educational charities and governmental bodies who are all stakeholders in this area. We are therefore concerned that much of the data around the progression of WP students in medicine might be open to criticism, especially if some of the definitions were used to make the category ever more inclusive, perhaps inflating the percentage of WP admissions to reach a target.

However, there is one group of medical applicants that is universally agreed upon as essential to include in any widening participation efforts: applicants over 21 years old with qualifications no higher than Level 3, e.g. secondary school AS-level (AS is the initial one-year component of a two-year-long A-Level qualification, taken by students typically aged 16–18).

These ‘mature non-graduates’, who left school without relevant qualifications or sufficient grades to enter medical school, and will have typically entered the labour market for a few years before returning to formal education, would first need to attend eligible Further Education colleges to obtain *Access to HE (Medicine)* diploma, or pass *A-levels* in the relevant subjects with the required grades as independent candidates, before applying to medicine. While they are relatively small in numbers, the group very likely represent genuine widening access students from educationally and socially disadvantaged backgrounds because of their non-standard pathway to medicine, after an interrupted education. Candidates from WP background face multiple barriers in applying for medicine, [21] and not all of these are likely to disappear if and when they gain entry to the course.

In this paper we set out to investigate whether the mature non-graduate students’ journey is comparable to that of the rest of the year cohort; whether they progress through the course non-inferiorly to school-leavers or graduate entrants, and if they are equally appointable to training posts after graduation. We created three groups – Group 1: school leavers; Group 2: graduate entrants who accessed medicine as a second degree; and Group 3: mature non-graduates, the group of interest.

Our aim was to (1) evaluate whether the dominance of prior academic qualifications in medical selection is fully justified, and (2) to follow the mature non-graduates through their education and training.

## Methods

### Cohort and statistics

A retrospective analysis was conducted to determine whether the progression of mature non-graduate students through the training pathway is comparable to that of the other two cohorts—school leavers and graduate entrants. We examined progression and non-completion rates during medical school, as well as career achievements post-graduation, to assess whether an attainment gap exists and persists throughout the course and into postgraduate training.

A comparative descriptive analysis was performed for each group using Kruskal-Wallis test, non-parametric statistical test to compare three independent groups, with Bonferroni correction as appropriate, and two-tailed Chi-square test. Logistic regression analysis controlling for demographic variables and prior academic attainment were used to analyse postgraduate ARCP (Annual Review of Competence Progression) outcomes.

Students’ on-course progression was assessed via the cumulative Educational Performance Measure (EPM) scores (score derived from a student’s ranking within their medical school cohort, which previously contributed to student’s overall score for allocating first-choice jobs in the UK Foundation Programme), and Pass/Fail in the compulsory Prescribing Safety Assessment (PSA).

The postgraduate measures analysed included scores from the ARCP, a formal, annual process used to assess medical trainees’ progression across Foundation, Specialty, and General Practice (GP) training programmes. By the end of Foundation training, newly qualified doctors apply to specialty training programmes in their chosen field and, for physician-led specialties, undertake the Membership of the Royal College of Physicians (MRCP) examinations (Parts 1 and 2, including PACES). These examinations are a core requirement for doctors pursuing careers in internal medicine and related medical specialties in the UK. [2, 22] Throughout training, doctors compile a comprehensive portfolio of evidence, including workplace-based assessments, documented clinical activities, performance achievements, supervisors’ appraisals, testimonials, and other forms of structured feedback. Trainees on the GP training programme are additionally required to sit the Royal College of General Practitioners Applied Knowledge Test (RCGP-AKT).

In addition, we compared the specialty choices of the three groups and analysed applications by the mature non-graduates to assess whether their ambition for clinical specialties is significantly different from their peers.

### Data

Data were provided by the University of Dundee Health Informatics Centre (HIC) on behalf of UKMED (UK Medical Education Database) and were collated for a total of 71,505 medical students who started the course between 2007 and 2015 (UKMEDP170), comprising of 55,000 school leavers (76.9%), 13,275 graduate students (18.6%) and 3,230 mature non-graduates (4.5%). Each participant was matched to include their demographic data as well as performance outcome data from undergraduate and postgraduate training, linking datasets from UCAS, UCAT, Higher Education Statistics Agency (HESA) and the Foundation Programme postgraduate recruitment and progression data (ARCP and MRCP). The cohorts were selected based on availability of data from the participants’ full journey, from application through to postgraduate training, to allow for meaningful data on their progression up to specialisation (data flowchart, Supplementary Table 1).

#### Variables and outcomes

Demographic variables that were considered in this study include: gender, ethnicity, disability, school type, parental higher education, socio-economic background as reported by attribution to a certain socio-economic classification (SEC), as well as index of multiple deprivation (IMD) and participation of local areas in higher education (POLAR). [23].

Outcome variables from prior to medical school entry included UCAT (University Clinical Aptitude Test), while on-course progression data were PSA, EPM, FP-SJT scores, and Primary Medical Qualification (PMQ) award number of years (i.e. the number of years spent obtaining PMQ). Postgraduate performance data consisted of ARCP, RCGP-AKT and MRCP examination results.

Although the PSA is not mandatory for graduation, high EPM scores were, until recently, essential for increasing the likelihood of securing a first-choice training post as a Foundation Year doctor.

Whereas there are different means to assess resident doctors’ progression against the benchmarks set by their training programme curriculum, we have chosen to focus our analysis on comparing the three groups of interest in the following ARCP Outcomes, both for Foundation and for Specialty training: [24, 25]

- Outcome 4, indicating that the trainee was released from the training programme, with or without specified competencies.
- Outcome 6: the trainee gained all required competencies and will be recommended as having completed the training programme (Core or Specialty).
- Developmental Outcome: Outcomes 2, 3, 4, 7.2, 7.3, RITA D or E – grouped together.

In addition, the success rate in passing the membership exams of the Royal Colleges at the first attempt (MRCP Part 1&2 and PACES; RCGP-AKT) was analysed for each group.

In terms of specialty recruitment, we analysed specialty applications to identify trends among the three groups of interest and examined application and offer rates for each group. Because it is possible that the trainee may not have accepted the position offered or may have been offered more than one place on the training programme, we have not included the acceptance rates. However, we did look at the recruitment to General Practice and compared the groups’ rates of applying exclusively to GP training, specifically at their first time of application.

## Results

The summary demographics in Table 1 show that the group of interest (mature non-graduates) differed in every measure of demographics we looked at: mature non-graduates trended with lower levels of parental higher education (compared with the school leavers), were from more disadvantaged backgrounds (based on socio-economic classification) and on average resided in less affluent neighbourhoods (judged by both IMD and POLAR). There were significantly higher percentages of males and those from Black and Minority Ethnic (BME) backgrounds among the mature non-graduate group than in the other two groups (*p* < 0.001). (The higher rates of missing data in the mature non-graduate group for these demographic variables must be noted however).

**Table 1:** Summary of demographic data for the three groups (*n* = 71505)

Outcome		School Leavers	Graduate Students	Mature non-graduates	Test of association	
					*Χ* ^*2*^	*p-value*
Gender	Male	24,295 (44.2%)	5930 (44.7%)	1680 (52.0%)	76.000	*< 0.001*
	Female	30,705 (55.8%)	7345 (55.3%)	1550 (48.0%)		
Ethnicity group	White	33,625 (61.1%)	9275 (69.9%)	1425 (44.1%)	949.490	*< 0.001*
	BME	19,150 (34.8%)	3320 (25.0%)	1550 (47.9%)		
	Missing	2225 (4.0%)	685 (5.2%)	260 (8.0%)		
Disability	No known disability	49,095 (89.3%)	11,145 (83.9%)	2740 (84.7%)	325.251	*< 0.001*
	One or more declared disability	5905 (10.7%)	2130 (16.1%)	495 (15.3%)		
School type	State school	34,020 (61.9%)	8135 (61.3%)	1415 (43.8%)	7939.317	*< 0.001*
	Privately funded	14,660 (26.7%)	695 (5.2%)	185 (5.8%)		
	Unknown/Not applicable	6315 (11.5%)	4445 (33.5%)	1630 (50.4%)		
Parental higher education	Yes	35,065 (63.8%)	7380 (55.6%)	1385 (42.8%)	895.568	*< 0.001*
	No	9640 (17.5%)	3020 (22.7%)	730 (22.6%)		
	Uknown	10,295 (18.7%)	2880 (21.7%)	1120 (34.6%)		
Socioeconomic classification (SEC)	5 - Managerial and professional occupations	32,195 (58.5%)	4615 (34.8%)	685 (21.2%)	5486.390	*< 0.001*
	4 - Intermediate occupations	4315 (7.8%)	1225 (9.2%)	235 (7.3%)		
	3 - Small employers and own account workers	2135 (3.9%)	235 (1.8%)	45 (1.5%)		
	2 - Lower supervisory and technical occupations	1010 (1.8%)	105 (0.8%)	35 (1.1%)		
	1 - Semi-routine and routine occupations	3680 (6.7%)	1770 (13.3%)	320 (9.9%)		
	Unknown	11,665 (21.2%)	5325 (40.1%)	1915 (59.2%)		
Index of multiple deprivation (IMD)	5 – least deprived	19,120 (34.8%)	3695 (27.2%)	480 (14.9%)	3079.534	*< 0.001*
	4	12,175 (22.1%)	2805 (21.1%)	400 (12.4%)		
	3	8555 (15.6%)	2310 (17.4%)	425 (13.2%)		
	2	5455 (9.9%)	1945 (14.7%)	350 (10.8%)		
	1 – most deprived	3665 (6.7%)	1085 (8.2%)	275 (8.5%)		
	Unknown	6030 (11.0%)	1435 (10.8%)	1230 (40.2%)		
Participation in local areas (POLAR)	5 – highest rate of participation	24,245 (44.1%)	5105 (38.5%)	675 (20.9%)	2798.975	*< 0.001*
	4	11,350 (20.6%)	2830 (21.3%)	440 (13.6%)		
	3	7095 (12.9%)	2095 (15.8%)	385 (11.9%)		
	2	4195 (7.6%)	1175 (8.9%)	265 (8.2%)		
	1 – lowest rate of participation	2065 (3.8%)	635 (4.8%)	170 (5.2%)		
	Unknown	6050 (11.0%)	1435 (10.8%)	1300 (40.2%)		

The table lists the three groups characterised by their demographic variables

Mature non-graduates had significantly poorer results in the cognitive component of UCAT (*p* = 0.000), and in all on-course progression outcome measures (*p* = 0.000) than the school leavers, but the gap was smaller when compared with the graduate student group (Table 2). They were outperformed by the graduates (*p* = 0.000) in UCAT-SJT and both of the cohorts performed better than the school leavers in this test (*p* = 0.000 and *p* = 0.002, respectively) (Table 2b). Mature non-graduates performed worse than the other two groups in PSA, EPM and FP-SJT (all *p* = 0.000) (Table 2), and took longer to obtain their primary medical qualification (PMQ) than either of the other groups (Table 3). The school leavers performed best in PSA and in FP-SJT.

**Table 2:** Summary outcome measures for pre-entry and on-course progression (mean)

Outcome		School Leaver	Graduate Student	Mature non-graduates	Test of difference	
					*Kruskal-Wallis H*	*p-value of significance*
UCAT	SJT Total	633.6 (*n =* 10075)	663.5 (*n =* 1080)	644.1 (*n =* 275)	267.274	*0.000*
	Cognitive Total	2626.8 (*n =* 52465)	2570.8 (*n =* 11615)	2508.4 (*n* = 2205)	801.886	*0.000*
PSA	Total	78.9 (*n =* 41645)	78.2 (*n =* 9105)	75.7 (*n =* 2050)	212.956	*0.000*
	Total Relative to Pass Mark	15.6 (*n =* 41645)	14.6 (*n =* 9105)	11.9 (*n =* 2050)	318.271	*0.000*
FP	EPM – Normal deviate	0.019 (*n =* 50100)	0.144 (*n =* 11655)	-0.125 (*n =* 2590)	291.086	*0.000*
	Foundation SJT (First attempt equated)	882.9 (*n =* 43605)	880.7 (*n =* 10435)	865.6 (*n* = 2260)	499.151	*0.000*

**Table 3:** Primary Medical Qualification award number of years for each group individually and in pairwise comparison

Outcome		School Leaver	Graduate student	Mature non-grads	Chi-sq	Significance
PMQ achieved from Medical School	PMQ achieved	92.0%(n=55000)	94.4%(n=13275)	87.7%(n=3230)	184.344	<.001
	PMQ in eight years	91.3%(n = 49090)	94.0%(n = 11565)	87.0%(n=2925)	173.380	<.001

PAIRWISE COMPARISON							
Outcome		School Leaver – Graduate student			School leaver – Mature non-grads		Graduate student – Mature non-grads
		Chi-sq	p-value^a^	Chi-sq	p-value^a^	Chi-sq	p-value^a^
PMQ achieved from Medical School	PMQ achieved	89.902	<.001	73.347	<.001	180.883	<.001
	PMQ in eight years	91.629	<.001	62.547	<.001	167.302	<.001

The success rate of mature non-graduates for MRCP Part 1 & 2, PACES and RCGP-AKT at first sitting was also inferior to the achievements of the school leaver group (*p* < *0.001*). In all four exams, the school leavers achieved the highest pass rates at first sitting, followed by graduates and finally mature non-graduates (Table 4).

**Table 4:** Postgraduate training outcome measures (passing MRCP exams and the ARCP Outcome results)

Outcome		School Leaver	Graduate student	Mature non-grads	Chi-sq	Significance
Membership exam	Passed MRCP part 1 (1st attempt)	70.1% (*n =* 11110)	61.9% (*n =* 2530)	57.2% (*n* = 580)	96.929	*< 0.001*
	Passed MRCP part 2 (1st attempt)	85.9% (*n* = 7055)	79.9% (*n =* 1540)	76.1% (*n* = 370)	54.500	*< 0.001*
	Passed MRCP PACES (1st attempt)	69.6% (*n =* 4550)	67.2% (*n* = 1075)	57.2% (*n* = 255)	18.743	*< 0.001*
	Passed RCGP-AKT (1st attempt)	89.4% (*n* = 6450)	82.6% (*n* = 2330)	76.5% (400)	112.279	*< 0.001*
ARCP Outcomes - ever	Outcome 4	0.8% (*n* = 45355)	1.5% (*n* = 12090)	1.9% (*n* = 2405)	70.358	*< 0.001*
	Developmental Outcome	8.4% (*n* = 45355)	13.6% (*n* = 12090)	17.7% (*n* = 2405)	472.146	*< 0.001*
	Outcome 6	86.1% (*n* = 45355)	89.1% (*n* = 12090)	84.9% (*n* = 2405)	83.326	*< 0.001*
ARCP Outcomes - Foundation	Outcome 4	0.1% (*n* = 45330)	0.2% (*n* = 12070)	0.4% (*n* = 2360)	20.839	*< 0.001*
	Developmental Outcome	1.4% (*n* = 45330)	2.0% (*n* = 12070)	3.1% (*n* = 2360)	55.641	*< 0.001*
	Outcome 6	85.9% (*n* = 45330)	88.9% (*n* = 12070)	84.7% (*n* = 2360)	83.167	*< 0.001*
ARCP Outcomes - Core	Outcome 4	2.2% (*n* = 12430)	4.0% (*n* = 3560)	4.4% (*n* = 685)	40.003	*< 0.001*
	Developmental Outcome	16.2% (*n* = 12430)	23.9% (*n* = 3560)	29.6% (*n* = 685)	168.722	*< 0.001*
	Outcome 6	50.1% (*n* = 12430)	47.8% (*n* = 3560)	45.8% (*n* = 685)	9.831	*0.007*
ARCP Outcomes - Specialty	Outcome 4	0.2% (*n* = 3785)	0.1% (*n* = 1100)	0.4% (*n* = 240)	1.346	*0.510*
	Developmental Outcome	8.2% (*n =* 3785)	13.7% (*n* = 1100)	17.1% (*n =* 240)	45.334	*< 0.001*
	Outcome 6	2.4% (*n* = 3785)	8.5% (*n =* 1100)	10.8% (*n* = 240)	111.430	*< 0.001*
ARCP Outcomes - GP	Outcome 4	0.1% (*n* = 8695)	0.1% (*n* = 3105)	0.5% (*n* = 545)	7.837	*0.020*
	Developmental Outcome	4.8% (*n* = 8695)	7.3% (*n* = 3105)	14.7% (*n* = 545)	105.715	*< 0.001*
	Outcome 6	49.2% (*n* = 8695)	51.7% (*n* = 3105)	51.5% (*n* = 545)	6.490	*0.039*

The table shows that fewer students from mature non-graduate group passed membership exams and had poorer outcomes in Annual Review of Competence Progression (ARCP)

Pairwise comparison for these outcomes is presented in Supplementary Table 2

As for training, approximately twice as many doctors from the mature non-graduate group accumulated Developmental Outcomes during the Foundation or Core training, compared to doctors from the school-leaver group, (ARCP Foundation, 3.1% vs. 1.4%, *p* < *0.001*; ARCP Core 29.6% vs. 16.2%; *p* < *0.001;* Table 4). Similarly, twice as many doctors from the mature non-graduate group had Outcome 4 recorded (released from the programme, with or without specified competencies: 4.4% vs. 2.2% (*p* < *0.001)* for Core or, 0.4% vs. 0.1% (*p* < *0.001)* for Foundation training; Table 3). As with the MRCP exams, Outcome 4 rates in Core and Foundation ARCP were significantly different when compared with the school leaver group, but not significantly different in pairwise comparison when mature non-graduates and the graduate students were compared (*p* = 0.666; *p* = 1.000).

Despite these rather wide attainment gaps, the percentages for Outcome 6 during the Core training look far more comparable between the three groups (50.1%, 47.8% and 45.8%, respectively, with the difference showing a lower level of significance at best in pairwise comparison (*p* = 0.042; *p* = 0.084 and *p* = 1.000). It is important to note that (possibly uneven) number of students from each of the three cohorts were still in Core training at the time of data collection and the final numbers for Outcome 6 are likely to be substantially higher.

The three groups also differed substantially in ARCP Outcomes for GP and Specialty training: 17.1% of doctors in the mature non-graduate group recorded a ‘developmental’ outcome during higher specialty training (but *n* was only 240), as opposed to 8.2% for the school leaver group, and 13.7% for the graduate student group *(p* < *0.001)*. Equally, 14.7% of ‘mature non-graduate’ doctors were reported to have a Developmental Outcome in GP training vs. 4.8% of school leavers and 7.3% of graduate groups, continuing the same ranking trend (Table 4). Nonetheless, this did not translate into lower Outcome 6 final percentages for mature non-graduates in GP training either: they had reasonably similar success rate as the other two groups during the GP training (*p* = 0.045; 0.906 and 1.000) and in fact, more of them achieved all the required competencies during Specialty training (Outcome 6: 10.8% vs. 2.4% (school leavers) vs. 8.5% (graduates), *p* < *0.001*, Table 4). As before, Outcome 6 for Specialty training is relatively low for any of the three groups, reflecting the fact that at the time of data collection many trainees had not yet completed the training and thus may not have *yet* recorded satisfactory completion of training; as the dataset matures and more up to date information is added to it, this limitation will likely diminish.

We have also analysed application and offer rates for specialty recruitments for each of the three groups (Table 5). A higher proportion of mature non-graduates appeared to apply to General Practice than the school leavers (*p* < *0.001*), however fewer did so exclusively (*p* = *0.846*;) and their offer rate was substantially lower (75.8% vs. 84.6%, *p* < *0.001*). In fact, offer rates in response to all applications (apart from Internal Medicine,*p* = 0.101) were statistically significantly lower for mature non-graduates than for the other two groups. It was also noted that a higher percentage of mature non-graduates applied for Core Surgical or Core Medical Training (both *p* < *0.001*) than either of the other two groups, but again, it was offset by the lower offer rates for these specialties (both *p* < *0.001*; Table 5).

**Table 5:** Specialty recruitment outcome measures

Outcome		School Leavers	Graduate students	Mature non-graduates	Chi-sq	Significance
Specialty recruitment	Applied – General Practice	37.3% (*n* = 33270)	43.6% (*n* = 9840)	45.6% (*n* = 1875)	165.057	< 0.001
	Exclusively GP Application (1st attempt)	24.0% (*n* = 33270)	25.4% (*n* = 9840)	22.9% (*n* = 1875)	10.967	0.004
	Applied – Core Anaesthetics Training	13.5% (*n* = 33270)	16.0% (*n* = 9840)	13.7% (*n* = 1875)	36.935	< 0.001
	Applied – Core Medical Training	16.3% (*n* = 33270)	16.8% (*n* = 9840)	21.7% (*n* = 1875)	38.146	< 0.001
	Applied – Internal Medicine Training	10.7% (*n* = 33270)	7.6% (*n* = 9840)	10.2% (*n* = 1875)	80.086	< 0.001
	Applied – Core Surgical Training	14.2% (*n* = 33270)	14.9% (*n* = 9840)	18.7% (*n* = 1875)	29.971	< 0.001
	Offered – General Practice	84.6% (*n* = 12405)	81.7% (*n* = 4295)	75.8% (*n* = 855)	56.957	< 0.001
	Offered – Core Anaesthetics Training	63.7% (*n* = 4505)	62.1% (*n* = 157)	54.1% (*n* = 255)	10.313	0.006
	Offered – Core Medical Training	80.8% (*n* = 5410)	73.8% (*n* = 1650)	71.7% (*n* = 405)	49.430	< 0.001
	Offered – Internal Medicine Training	80.8% (*n* = 3550)	77.7% (*n* = 750)	77.5% (*n* = 190)	4.587	0.101
	Offered – Core Surgical Training	59.6% (*n* = 4725)	55.6% (*n* = 1470)	48.3% (*n* = 350)	22.005	< 0.001

The table shows which specialties each of the three groups have applied to, and the training pathways they were offered

The data suggests that mature non-graduates are more likely to apply for more than one specialty training programme, perhaps indicating less confidence that they would be accepted, which appears to be borne out by the data presented here as the mature non-graduates received the lowest acceptance rates across the board, particularly compared to the school leaver group.

## Discussion

This is the first study that looked at mature non-graduates’ progression across the entire medical training pathway using data from the UKMED, including 71,505 medical students in the analysis. We further investigated specialty choices and acceptance rates in this cohort, adjusting for confounding factors that may have influenced career decision-making. The result is a complete overview of the performance of mature non-graduate students from the start to the finish of their training in UK medical schools, measured against their contemporary peers from the school leaver and graduate student entry groups.

While one prior study has reported on the relative success of students from widening participation backgrounds entering via Gateway courses, [11] this is the first comprehensive comparative analysis to examine the educational and professional outcomes for students who came into Medicine via Further Education colleges, attempting to analyse the trend this subset of widening participation students/doctors show in selecting and finally embarking on the training pathway. This group of students, having had an interrupted education, are older than the school leavers and less academically qualified than entrants from either group. Our data show that these academic differences persist throughout their education and training, both in pre-entry and the foundation program selection. Interestingly, the one measure in which the mature non-graduates and graduates both do better than the school leavers is the UCAT SJT, which could be said to be an assessment of maturity rather than academic prowess; indeed, it was the only test in which the school leavers performed statistically significantly worse than both of the mature student groups.

Although for all measures of academic attainment a stronger performance was seen with the school leavers group, and the differences seen here are highly significant because of the large cohort sizes, they cannot be said to be dramatic in magnitude. Nonetheless, the outcome differences are generally larger when mature non-graduates are compared to the school leavers, rather than to the graduate students’ group. The academic differences persist in all medical school outcomes, and in most postgraduate outcomes too, particularly the ones related to exams, such as first pass rate in MRCP, PACES and RCGP-AKT. These high-stakes, summative exams are essential to confirm that a doctor has the knowledge, skills, and judgement required for safe, independent practice [26] and passing them is essential for obtaining a Certificate of Completion of Training and joining the professional register, enabling unsupervised practice in the UK.

As for ARCP outcomes, mature non-graduates continue to show some disadvantage (inferior ‘Outcome 4’ and ‘Developmental Outcome Ever’ rates, Table 4), but from the as yet incomplete and premature data, they appear to complete training at a similar rate as the other two groups, judged by similar Outcome 6 scores (including ‘Outcome 6 for GP training’), even though they take much longer to graduate (PMQ number of years, Table 3). Our data show that the success rate of mature non-graduates is closer to that of graduate students than to that of school leavers across almost all measures examined in this study (EPM being the main exception). It is plausible that life commitments had a negative impact on progress in this group, but lacking data, this was not further explored.

The annual review is an important measure of the resident doctor’s progress, but it is also an interim measure of their readiness for practice. Mature non-graduates are more likely to struggle with postgraduate training (as Outcome 4 rates suggest). They are older than the school leavers and may have greater family or financial commitments during this time, all of which could negatively affect their review outcomes. Their longitudinal assessments are likely to have been impacted by personal circumstances and possibly by a lack of ‘know-how’ in navigating the system. While our findings do not indicate that this group achieves parity with their peers in educational or training outcomes, nor support the notion that a latent pool of capable candidates is excluded by the current selection system, this study demonstrates that students in mature non-graduate group do graduate and complete medical training, albeit at slightly lower—yet still comparable—rates to their peers, thereby justifying the current admissions policies pertaining to this group. They appear to complete training at rates comparable to others, qualify as doctors, and make meaningful contributions to the healthcare workforce. Irrespective of this positive conclusion, widening participation in medicine is not, and has never been, justified solely by performance outcomes, but by fundamental principles of social justice and equity.

The other important message from this study is the career choice trend among mature non-graduates – they seem to pursue similar training pathways as the other two groups, and we find no evidence that mature non-graduates particularly favour a career in General Practice. Our analysis shows that while more mature non-graduates apply to GP training, they are significantly less likely to exclusively apply for it than the other two groups. When mature non-graduates apply for GP training - at a slightly higher rate than school leavers — around half of them concurrently apply for a training place in another specialty too. In contrast, school leavers applying for GP training are less likely to submit concurrent applications to other specialties, and a substantially higher proportion of their applications are successful (84.6% compared with 75.8% for mature non-graduates). A reasonable interpretation of these data is that students in the school leavers group are more confident in securing their first-choice training placements, and we find that this confidence is well-founded. The fact that mature non-graduates’ application rates for more competitive training programmes yield lower ‘offer’ rates, raises the question of equity in the postgraduate arena too [27] and more research is needed to understand the causes of these poor acceptance rates.

Our findings are consistent with those of Gale et al., [15] who reported that 43% of their sample applied to GP specialty training, although only 26% listed it as their sole specialty choice. Their study identified a strong correlation between a doctor’s socio-economic background and the decision to pursue General Practice. Given that the paper was published nearly a decade ago, the landscape appears to have evolved. One important development is that today’s generation of widening participation doctors are notably more ambitious, increasingly willing to forge their own paths, and determined to enter specialties that were previously perceived as inaccessible to them.

We think that the topic of this study is of huge interest to selectors in the UK as previous research suggested that students from widening participation background often prefer to work as GPs in underserved areas. [28, 29] It is possible that the situation has changed over time. The evidence presented here does not exactly contradict published reports but does suggest that the ambitions of resident doctors that started medical education as mature non-graduates is not markedly different from those of their peers, despite the fact that they perform less well on average during both undergraduate and postgraduate training. This does not appear to deter them from applying to highly competitive specialties, albeit sometimes alongside General Practice, which has also become a sought-after career destination in recent years. Further evidence is needed to determine whether non-standard pathways to higher education ultimately act as a leveller - driven by the resilience and life experience that prior work can provide - or whether they represent a persistent constraint on doctors’ ambition, progression, and inclusion.

## Conclusions

Contextual admissions for students from disadvantaged backgrounds should be maintained, as such approaches do not “lower the bar” but instead enable academic achievement to be interpreted within the context of prior opportunity. Success in the presence of socioeconomic and educational disadvantages may reflect attributes such as resilience, adaptability, and sustained motivation - qualities that are essential for medical training and practice. Consistent with this interpretation, our study demonstrates that ultimately the subset of students we looked at that were admitted through widening participation routes, perform at a level comparable to their peers from other admission pathways, despite having experienced a longer and more complex educational trajectory. The focus should therefore shift from whether contextual admissions should exist or not to how these students can be supported effectively in undergraduate and postgraduate worlds to ensure both equity and excellence in medical education.

## Supplementary Information

Supplementary Material 1.

Supplementary Material 2.

## Data Availability

Source - UK Medical Education Database (“UKMED”) UKMEDP170 extract first generated on 03/01/2024 and approved for publication on 5/12/2025. We are grateful to UKMED for the use of this data. However, UKMED bears no responsibility for our analysis or interpretation. The data includes information derived from that collected by the Higher Education Statistics Agency Limited (“HESA”) and provided to the GMC (“HESA Data”). Source: HESA Student Record 2007 to 2015 Copyright Higher Education Statistics Agency Limited. The Higher Education Statistics Agency Limited makes no warranty as to the accuracy of HESA Data, cannot accept responsibility for any inferences or conclusions derived by third parties from data or other information supplied by it. Applications to view the data can be made to UKMED. The data in UKMED are anonymised before use and the GMC acts as data controller ensuring the security of the data and compliance with the data protection laws (more detail can be found here: UK Medical Education Database - GMC). We are not permitted to share raw data with other parties, without the advance written permission from the GMC.

## Abbreviations

ARCP: Annual Review of Competence Progression
BME: Black and Minority Ethnics
CAT: Core Anaesthetics Training
CMT: Core Medical Training
CST: Core Surgical Training
EPM: Educational Performance Measure
HESA: Higher Education Statistics Agency
IMD: Index of Multiple Deprivation
