# Supplementary material for: How do mature non-graduate students compare to the rest of the cohort in medical training? A UKMED study
Source: BMC Med Educ. 2026 Jun 26;26:1617. doi: 10.1186/s12909-026-09776-6 (PMC13656443; doi:10.1186/s12909-026-09776-6)
Supplement: Supplementary file 1 [file 12909_2026_9776_MOESM2_ESM.pdf]

Supplementary table 2. Postgraduate training outcome measures (peerwise comparison, to complement Table 4)

Outcome		*School Leaver – Graduate student*		School leaver – Mature non-grads		Graduate vs Mature non-grads	
		Chi-sq	***p-value***[a]	***Chi-sq***	***p-value***[a]	***Chi-sq***	***p-value***[a]
**Membership exam**	Passed MRCP part 1 (1st attempt)	64.400	**<.001**	43.103	**<.001**	4.301	*0.114*
	Passed MRCP part 2 (1st attempt)	34.765	**<.001**	26.798	**<.001**	2.630	*0.315*
	Passed MRCP PACES (1st attempt)	2.460	0.351	17.532	**<.001**	9.081	**0.009**
	Passed RCGP AKT (1st attempt)	72.667	**<.001**	62.235	**<.001**	8.507	**0.012**
**ARCP Outcomes - ever**	Outcome 4	51.147	**<.001**	32.427	**<.001**	1.934	*0.492*
	Unsatisfactory outcome	302.108	**<.001**	249.334	**<.001**	28.563	**<.001**
	Outcome 6	76.876	**<.001**	2.767	0.288	35.404	**<.001**
**ARCP Outcomes - Foundation**	Outcome 4	12.276	**<.001**	13.769	**<.001**	1.489	*0.666*
	Unsatisfactory outcome	22.427	**<.001**	42.111	**<.001**	10.501	**0.003**
	Outcome 6	77.040	**<.001**	2.580	0.324	34.453	**<.001**
**ARCP Outcomes - Core**	Outcome 4	33.221	**<.001**	12.943	**<.001**	0.219	*1.000*
	Unsatisfactory outcome	111.352	**<.001**	82.846	**<.001**	9.969	**0.006**
	Outcome 6	6.039	**0.042**	4.844	0.084	0.905	*1.000*
**ARCP Outcomes - Specialty**	Outcome 4	0.271	1.000	0.867	1.000	1.402	*0.711*
	Unsatisfactory outcome	31.102	**<.001**	22.648	**<.001**	1.793	*0.543*
	Outcome 6	88.734	**<.001**	58.548	**<.001**	1.368	*0.726*
**ARCP Outcomes - GP**	Outcome 4	0.070	1.000	6.900	**0.027**	5.801	**0.048**
	Unsatisfactory outcome	27.385	**<.001**	98.025	**<.001**	32.846	**<.001**
	Outcome 6	5.952	**0.045**	1.065	0.906	0.014	*1.000*

a. Significance values have been adjusted by the Bonferroni correction for multiple tests.

The tree groups postgraduate outcomes are shown in peerwise comparison – mature non-graduates compare poorly to the school-leaver group but are closer in performance to the graduate group
